# Long non‐coding RNA MEG3 promotes autophagy and apoptosis of nasopharyngeal carcinoma cells *via* PTEN up‐regulation by binding to microRNA‐21

**DOI:** 10.1111/jcmm.15759

**Published:** 2020-12-17

**Authors:** Liqiang Lin, Xiaoli Liu, Baotao Lv

**Affiliations:** ^1^ Otolaryngological Department Linyi People’s Hospital Linyi PR China; ^2^ Psychology Department Linyi Rongjun Hospital Linyi PR China; ^3^ Radiology Department Linyi People’s Hospital Linyi PR China

**Keywords:** apoptosis, autophagy, maternally expressed gene 3, microRNA‐21, nasopharyngeal carcinoma, phosphatase and tensin homologue

## Abstract

Long non‐coding RNAs (lncRNAs) have been highlighted as attractive markers for diagnosis and prognosis as well as new therapeutic targets in multiple cancers, including nasopharyngeal carcinoma (NPC). Here, we attempted to investigate the underlying regulatory role of the lncRNA maternally expressed gene 3 (MEG3) in NPC development. As determined by RT‐qPCR, MEG3 expression was down‐regulated in NPC cells. Online RNA crosstalk analysis predicted the binding of miR‐21 to MEG3 and PTEN, respectively. MEG3 was validated to bind to miR‐21 while PTEN was identified as a target of miR‐21 by dual‐luciferase reporter gene assay. Exogenous transfection was done to change the levels of MEG3, miR‐21 and PTEN in HK‐1 cells to investigate their effects on the autophagy and apoptosis of NPC cells. The results suggested that MEG3 overexpression in HK‐1 cells up‐regulated PTEN and down‐regulated miR‐21, by which MEG3 further inhibited autophagy and apoptosis ability of NPC cells. The tumour formation ability was tested after injecting the HK‐1 cells into nude, mice and tumour growth was monitored. Consistently, MEG3 overexpression inhibited the tumour formation in vivo. Collectively, MEG3 promotes the autophagy and apoptosis of NPC cells via enhancing PTEN expression by binding to miR‐21.

## 1 INTRODUCTION

Nasopharyngeal carcinoma (NPC) is the most peculiar head and neck cancer given that its geographic and ethnic distribution varies distinctly. The dynamic interplay of multifactorial aetiology, including Epstein‐Barr virus infection, environmental carcinogens and genetic predisposition are considered as the major players in its aetiology.^1^, ^2^ Although to a large degree, more precise approaches of imaging and radiotherapy aid in achieving the successful local control of NPC, however, distant metastases remained challenging for NPC treatment.^3^ Various long non‐coding RNAs (lncRNAs) involve in regulating apoptosis of NPC^4^ revealing lncRNAs as promising diagnostic markers and therapeutic targets of NPC.

Moreover, LncRNAs have been widely reported to modulate the tumour initiation, growth and metastasis as they can regulate the chromatin organization, transcription and post‐transcription by interacting with protein molecules, DNA and RNA.^5^ Particularly, lncRNA maternally expressed gene 3 (MEG3) has been reported as an imprinted gene on DLK1‐MEG3 locus in human chromosome 14q32.2.^6^ MEG3 expresses poorly in various primary human tumours and tumour cell lines, and MEG3 inhibits proliferation of tumour cells.^7^ Notably, ectopic expression of MEG3 leads to cell cycle arrest, and inhibition of colony formation, and cell proliferation in NPC.^8^ Our prediction revealed that MEG3 could bind to a small non‐coding RNA molecule microRNA‐21 (miR‐21). miRNAs are well known to inhibit their target gene expression in a sequence‐dependent manner, therefore, functioning in diverse cellular processes of cancers.^9^, ^10^ Although, overexpressed miR‐21 is associated with the progression of various cancers including NPCs,^11^, ^12^ scarce data on the downstream targets of miR‐21 in NPCs have been obtained. Of note, our sequence analysis and mRNA‐miRNA interaction prediction revealed that phosphatase and tensin homologue (PTEN) is a potential target gene of miR‐21. Moreover, PTEN is one of the tumour suppressor genes with the most frequently inactivated expression in sporadic cancer.^13^ Interestingly, the down‐regulation of PTEN occurring in NPC cells, promotes the progress of tumour cell growth, migration and invasion.^1^ Based on these aforementioned findings, we have been suggested an interaction between MEG3, miR‐21 and PTEN, which may participate in the biology of NPCs.

## 2 METHODS AND MATERIALS

### 2.1 Ethics statement

The study protocol was approved by the Ethics Committee and Experimental Animal Ethics of Linyi People's Hospital. Informed written consent was obtained from each patient before the study. All the experiments were conducted strictly in accordance with the Helsinki Declaration. The animal experiment strictly adhered to the principle to minimize the pain, suffering and discomfort to experimental animals.

### 2.2 Study subjects

Fresh frozen NPC clinical tissue specimens and corresponding paracancerous tissue specimens were collected from 80 NPC patients who received operations in Linyi People's Hospital from December 2015 to November 2018. All these patients were followed up for 3 years. Four NPC cell lines (C666‐1, HK‐1, 5‐8F and 6‐10B) and nasopharyngeal epithelial cell line (NP69) from National Infrastructure of Cell Line Resource (http://www.cellresource.cn/) were cultured in complete DMEM (Gibco) with 5% CO_2_ and 95% saturated humidity at 37℃. Cells were passaged after reaching 90% confluence.

### 2.3 Cell transfection

In the present study, NPC cells were transfected with overexpressed (oe)‐negative control (NC), oe‐MEG3, small hairpin RNA (sh)‐NC, sh‐MEG3, NC inhibitor, miR‐21 inhibitor, NC mimic, miR‐21 mimic, miR‐21 mimic + oe‐NC, miR‐21 mimic + oe‐MEG3, oe‐NC + sh‐NC, oe‐MEG3 + sh‐NC and oe‐MEG3 + sh‐PTEN in combination, respectively. All the plasmids or sequences were from Dharmacon. Cells were inoculated into a 6‐well plate with cell density reaching 80%‐ 90%, and then, transfection was conducted using the lipofectmine 2000 kit (Invitrogen). The medium was renewed 8 hours after transfection. Subsequent experiments were performed after 48 hours of transfection.

### 2.4 Reverse transcription–quantitative polymerase chain reaction (RT‐qPCR)

The total RNA was extracted and then reversely transcribed into cDNA. Then, the sample was loaded, followed by real‐time qPCR in the ABI7500 qPCR instrument (ABI) using the SYBR Premix EX Taq kit (RR420A, Takara). Three replicate wells were set for each sample. All primers were synthesized by Shanghai Sangon Biotechnology Co. Ltd. (Table 1). The Ct value of each well was recorded with glyceraldehyde‐3‐phosphate dehydrogenase (GAPDH) or U6 used as an internal reference. The relative expression of the RNA of interest was calculated using the 2‐ΔΔCt method.

**Table 1:** Primer sequences for RT‐qPCR

Gene	Primer sequences
MEG3	F:5'‐CTGCCCATCTACACCTCACG‐3'
	R:5'‐CTCTCCGCCGTCTGCGCTAGGGGCT‐3'
miR‐21	F:5'‐ACTCTAGAGTCGACACCACTGACTATGATC‐3'
	R:5'‐ACTCTAGACATGACACAGCTACACAACC‐3'
PTEN	F:5'‐GGACGAACTGGTGTAATGATATG‐3'
	R:5'‐TCTACTGTTTTTGTGAAGTACAGC‐3'
LC3II	F: 5′‐CCGACCGCTGTAAGGAGGTA‐3′
	R: 5′‐AGGACGGGCAGCTGCTT‐3′
Beclin I	F: 5′‐TGTCACCATCCAGGAACTCA‐3′
	R: 5′‐CTGTTGGCACTTTCTGTGGA‐3′
P62	R:5'‐TGATGCTGGTGCTGAGTATGT‐3'
	R:5′‐AGAATGGGAGTTGCTGTTGAAGT‐3′
GAPDH	F:5'‐ACCACCATGGAGAAGGCTGG‐3'
	R:5'‐CTCAGTGTAGCCCAGGATGC‐3'
U6	F:5'‐GCTTCGGCAGCACA‐3'
	R:5'‐AACGCTTCACGAATTTGCGT‐3'

Note

RT‐qPCR, reverse transcription–quantitative polymerase chain reaction; MEG3, maternally expressed gene 3; miR‐21, microRNA‐21; PTEN, phosphatase and tensin homologue; GAPDH, glyceraldehyde‐3‐phosphate dehydrogenase; F, forward; R, reverse.

### 2.5 Fluorescence in situ hybridization (FISH)

The subcellular localization of MEG3 was identified by FISH. Following the manufacturing instructions of Ribo^TM^ lncRNA FISH Probe Mix (Red) (C10920, RiboBio Co., Ltd., Guangzhou), the cells (6 × 10^4^ cells/well) were inoculated in a 24‐well culture plate to reach 60%‐70% confluence. Subsequently, the cells were fixed for 10 minutes using 1 mL of 4% paraformaldehyde at room temperature and permeabilized with 1 mL pre‐cooled phosphate buffer saline (PBS) containing 0.5% Triton X‐100 at 4℃ for 5 minutes. Then, 200 μL pre‐hybrid solution was added and blocked at 37℃ for 30 minutes. The pre‐hybrid solution was replaced by a hybridization solution containing probe (anti‐MEG3 nucleotide probe, GeneCreate Biological Engineering Co., Ltd., Hubei, China) to hybridize at 37℃ in the dark overnight. The washing solution I [4 × systemic sclerosis (SSC), 0.1% Tween‐20), washing solution II (2 × SSC), washing solution III (1 × SSC) and 1 × PBS at 42℃ were successively added. Thereafter, the cells were stained by 4'‐6‐diamidino‐2‐phenylindole (DAPI) staining solution (1:800) for 10 minutes and sealed by nail polish after rinsing in the washing solution. Five different fields of view were selected under a fluorescence microscope (Olympus Optical Co., Ltd).

### 2.6 RNA immunoprecipitation (RIP)

Cells were lysed with RIP Lysis Buffer (N653‐100 mL, Shanghai Haoran Biotechnologies Co., Ltd.) for 5 minutes on ice. A total of 50 μl magnetic beads was then added into each tube and then added with 0.5 mL RIP Wash Buffer (EHJ‐BVIS08102, Xiamen Huijia Biotechnology Co., Ltd.) to aggregate magnetic beads on the magnetic separator after a brief vortex. After that, 100 μl RIP Wash Buffer and 5 μg Ago2 antibody (P10502500, Shenzhen Otwo Biotech Inc, Guangdong, China) were added. Goat anti‐rabbit Immunoglobulin G (IgG) secondary antibody (as NC) was added and incubated for 30 minutes. The magnetic bead‐antibody complex together with 900 μl of RIP Buffer (P10403138, Shenzhen Otwo Biotech Inc) was centrifuged at 4℃ for 10 minutes. The supernatant was aspirated into a new Eppendorf tube (LBCT015S, Beifang Tongzheng Biotechnology Co., Ltd). A total of 100 μl of the supernatant was added into a tube with the magnetic bead‐antibody to make a final immunoprecipitation reaction volume of 1.0 mL and incubated at 4℃ overnight. After that, the magnetic beads were washed with 0.5 mL RIP Wash Buffer. A total of 150 μl proteinase‐K buffer was added for incubation at 55℃ for 30 minutes to purify the RNA and then RNA was extracted using the conventional TRIzol method and then subjected to RT‐qPCR.

### 2.7 Western blot analysis

The total protein in tissues or cells was isolated using RIPA lysis buffer containing phenylmethylsulphonyl fluoride. Subsequently, the protein was dissolved in 2 × sodium dodecyl sulphate (SDS) loading buffer and subjected to SDS‐PAGE gel electrophoresis. The protein was then transferred to polyvinylidene fluoride membrane by wet transfer method and blocked with 5% skim milk powder for 1 h. After that, the PVDF membrane was incubated with diluted primary anti‐human rabbit phosphatase and tensin homologue (PTEN) (ab170941, 1:100), rabbit anti‐human Bax (ab32503, 1:1000), rabbit anti‐human Bcl‐2 (ab32124, 1:1000), rabbit anti‐human P62 (ab56416, 1:1000), rabbit anti‐human Beclin1 (ab62557, 1:1000), rabbit anti‐human LC3II (ab48394, 1:1000) and Cleaved‐Caspase3 (ab13585, 1:1000) at 4℃ overnight, respectively. Rabbit anti‐human GAPDH (ab9485, 1:2500) was used as the internal reference. All antibodies used above were from Abcam (Cambridge, UK). After being washed with tris‐buffered saline with Tween 20 (TBST), the membrane was incubated together with horseradish peroxidase (HRP)‐labelled secondary anti‐goat anti‐rabbit IgG H&L (ab97051, 1:2000, Abcam, Shanghai China) for 1 h and placed on a clean glass plate. The membrane was developed using an enhanced chemiluminescence kit (Cat. No. BB‐3501, Amershame, UK) and photographed using a Bio‐Rad image analysis system (Bio‐Rad) and analysed by QuantityOnev 4.6.2 software.

### 2.8 Dual‐luciferase reporter gene assay

The reporter plasmids wt‐MEG3, mut‐MEG3, wt‐PTEN and mut‐PTEN were designed by GenePharma. NC mimic and miR‐21 mimic were cotransfected with wt‐MEG3, mut‐MEG3, wt‐PTEN and mut‐PTEN into HK‐1 cells, respectively, and the transfected cells were collected after incubation for 48 hours. The luciferase activity was measured following the instructions provided by Genecopoeia's dual‐luciferase assay kit (D0010, Beijing Solabio Life Sciences Co., Ltd,). The luminance was detected using a Promega GLomax 20/20 Luminometer (E5311, Zhongmei Biotechnology Co., Ltd.).

### 2.9 Immunofluorescence assay

Cells were fixed and blocked by 3% bovine serum albumin, which was followingly incubated overnight with rabbit anti‐LC3II (ab48394, 1:1000, USA) at 4℃. Subsequently, cells were incubated with red fluorescent secondary antibody (Intertek) for 2 hours and sealed after staining for 5 minutes by DAPI (1 μg/mL), followed by observation and photographing under a fluorescence microscope (Olympus Optical Co., Ltd).

### 2.10 TdT‐mediated dUTP‐biotin nick end‐labelling (TUNEL) staining

The apoptosis of HK‐1 cells was analysed by TUNEL staining. HK‐1 cells were cultured as described previously, trypsinized and fixed in 1% paraformaldehyde in PBS at a concentration of 1 × 10^6^ cells/mL. Next, the cells were resuspended overnight in frozen ethanol at −20℃ and centrifuged the next day. Subsequently, cells were treated with terminal deoxynucleotidyl transferase and fluorescein in combination with deoxyuridine triphosphate, after which cells were incubated in pi/RNase staining buffer. The cells were observed under the Axiovert fluorescence microscope after the nuclei were stained with DAPI.

### 2.11 Flow cytometry

The Annexin‐V‐fluorescein isothiocyanate (FITC), phosphate (PI) and \2‐[4‐(2‐hydroxyethyl)‐1‐piperazinyl]ethanesulphonic acid (HEPES) buffer solution were formulated into Annexin‐V‐FITC/PI dye solution at a ratio of 1:2:50 according to the Annexin‐V‐FITC Apoptosis Detection Kit directions (K201‐100, Biovishon). Each 100 μl staining solution was used to resuspend 1 × 10^6^ cells. After incubation for 15 minutes, 1 mL HEPES buffer (PB180325, Procell) was added. Subsequently, 525 and 620 nm band‐pass filters were excited at 488 nm to detect FITC and PI fluorescence and to assess the apoptosis of cells.

### 2.12 Xenograft tumour in nude mice

A total of 24 BALA/c nude mice (aged 4 weeks, weighing 18‐ 25 g) were purchased from Guangdong Experimental Animal Center (Guangdong, China) (http://www.gdmlac.com.cn/) and fed in a specific pathogen‐free environment. These mice were subcutaneously injected with 1 × 10^6^ HK‐1 cells and stably transfected with oe‐NC and oe‐MEG3. The weight and volume of xenograft tumours were recorded on the 7th, 14th, 21st and 28th days after inoculation using V = (A × B2)/ 2 (mm^3^, A, the long diameter, B, the short diameter). The graph of the average volume at each time point was plotted. The nude mice were killed by carbon dioxide asphyxiation to collect the tumour tissues.

### 2.13 Immunohistochemistry

After heating at 60℃ for 1 hour, tissue sections were dewaxed by conventional xylene, hydrated with gradient ethanol and incubated with 0.5% Triton in PBS for 20 minutes. Subsequently, the tissue sections were subjected to high‐pressure antigen retrieval for 2 minutes and then boiled in a 0.01 M citrate buffer with a pH of 6.0 at 95℃ for 20 minutes. The sections were immersed in 3% H_2_O_2_ for 15 minutes to block the exogenous peroxidase activity and then blocked with 3% bovine serum albumin and gently shaken at 37℃ for 20‐30 minutes. The tissue sections were then incubated with diluted primary antibody: rabbit anti‐human PTEN (ab170941, 1:100), rabbit anti‐human Bax (ab32503, 1:250), rabbit anti‐human Bcl‐2 (ab32124, 1:250), rabbit anti‐human P62 (ab56416, 1:1000), rabbit anti‐human Beclin1 (ab62557, 1:200) at 37℃ for 2 hours. All antibodies used above were purchased from Abcam. The tissue sections were added with 1:1000 diluted HRP‐labelled goat anti‐rabbit IgG secondary antibody (ab6721, Abcam, Cambridge, UK) and incubated in a humid box at 37℃ for 30 minutes. Haematoxylin (Shanghai Fusheng Industrial Co., Ltd.) was used to counterstain the tissue sections for 4 minutes. The tissue sections were then sealed by 10% glycerol and observed under the microscope. The results of immunohistochemistry were scored in a double‐blind manner.

### 2.14 Statistical analysis

SPSS 21.0 statistical software (IBM Corp.) was used for data analysis. The measurement data were described as mean ± standard deviation. A paired t test was used to compare the two sets of data with normal distribution and homogeneity of variance, while an unpaired t test was used to test two sets of unpaired data with normal distribution and homogeneity of variance. Data among multiple groups were compared by one‐way analysis of variance (ANOVA) and Tukey's post hoc tests. The data of different groups at different time points were compared by repeated‐measures ANOVA and Bonferroni's post hoc tests. The difference was regarded as statistically significant when *P* < .05.

## 3 RESULTS

### 3.1 MEG3 is poorly expressed in NPC tissues

**FIGURE 1:**
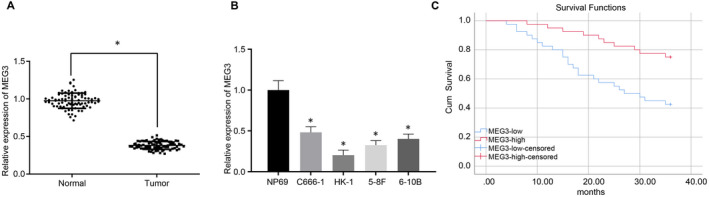
The lower expression of MEG3 is associated with the poor prognosis of NPC. A, Relative expression of MEG3 in NPC tissues and paracancerous tissues measured by RT‐qPCR. B, Relative expression of MEG3 in normal nasopharyngeal epithelial cell line NP69 and four NPC cell lines (C666‐1, HK‐1, 5‐8F and 6‐10B) measured by RT‐qPCR. C, Kaplan‐Meier curve indicates the correlation between the MEG3 expression and the prognosis of patients with NPC. And the survival difference of patients is shown by the log‐rank analysis. * indicates *P* < .05 compared to paracancerous tissues or normal nasopharyngeal epithelial cells. The measurement data were described as mean ± standard deviation. A paired t test and one‐way ANOVA were used in the comparison of two sets of data or data among multiple groups, respectively. In panel C, the overall survival of patients was described as the percentage through the Kaplan‐Meier, and the log‐rank analysis was used to calculate the *p*‐value

To verify the results in the previous study that MEG3 was down‐regulated in NPC,^8^ RT‐qPCR was performed to measure the expression of MEG3 in 80 pairs of NPC tissues and paracancerous tissues. The expression of MEG3 in NPC tissues was lower than that in the paracancerous tissues (*P* < .05) (Figure 1A). Consistently, MEG3 was poorly expressed in four NPC cell lines (C666‐1, HK‐1, 5‐8F and 6‐10B), compared with one normal nasopharyngeal epithelial cell line NP69 (Figure 1B). As HK‐1 cells exhibited the lowest MEG3 level among the four NPC cell lines, this cell line was used for the subsequent cell experiments. Interestingly, Kaplan‐Meier curve analysis showed that low expression of MEG3 was associated with poor prognosis in patients with NPCs (Figure 1C).

### 3.2 MEG3 overexpression promotes the apoptosis and autophagy of NPC cells

**FIGURE 2:**
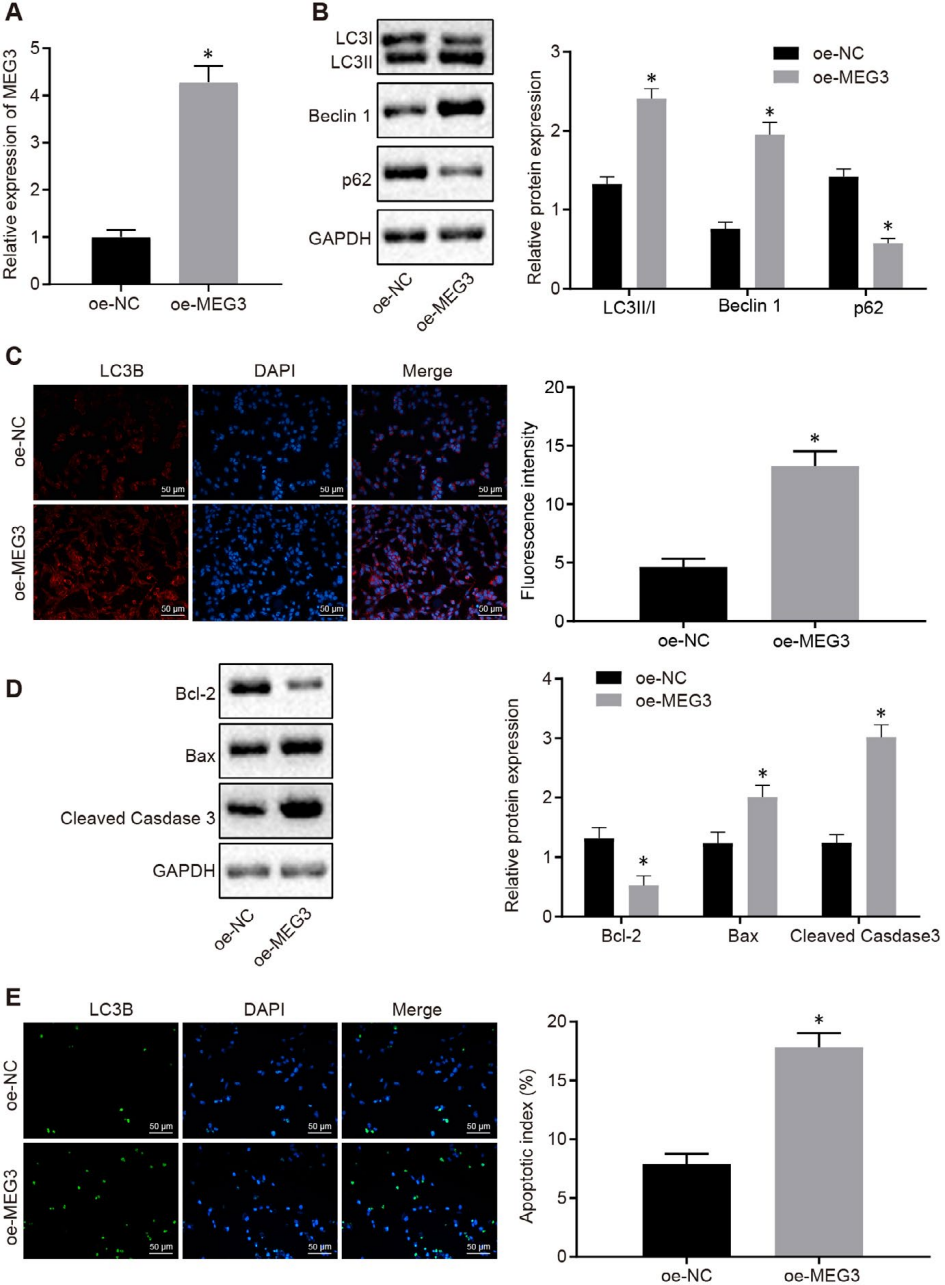
MEG3 overexpression enhances the apoptosis and autophagy of NPC cells. A, The relative level of MEG3 in the HK‐1 cells transfected with oe‐NC and oe‐MEG3 by RT‐qPCR. B, Western blot analysis to quantify the levels of autophagy‐related proteins in the HK‐1 cells transfected with oe‐NC and oe‐MEG3. C, Immunofluorescence assay to monitor the expression of LC3II (×200) in the HK‐1 cells transfected with oe‐NC and oe‐MEG3. D, Western blot analysis of the expression of the apoptotic‐related proteins in the HK‐1 cells transfected with oe‐NC and oe‐MEG3. E, TUNEL staining to count the apoptotic HK‐1 cells after transfection with oe‐NC and oe‐MEG3 (×200). The data were described as mean ± standard deviation. The difference was statistically significant when *P* < .05. Independent sample t test was used to compare data between two groups, while one‐way ANOVA and Tukey's post hoc tests were applied for data comparison among multiple groups. Three independent experiments were performed

NPC cells (HK‐1) were transfected with oe‐MEG3 and oe‐NC, respectively. MEG3 was detected at a higher expression in cells transfected with oe‐MEG3 than the oe‐NC‐transfected cells, validating the successful transfection (Figure 2A).

Western blot analysis showed that compared with oe‐NC‐transfected cells, increased expression of autophagy‐related protein LC3II and Beclin1, and decreased expression of p62 were observed in cells transfected with oe‐MEG3 (*P* < .05) (Figure 2B). Consistent with the immunoblotting data, immunofluorescence assay detected a higher level of LC3II in oe‐MEG3‐transfected cells than that in oe‐NC‐transfected cells (*P* < .05) (Figure 2C).

MEG3 overexpression caused a decreased expression of Bcl‐2, an anti‐apoptosis protein as well as up‐regulated the expression of pro‐apoptosis protein Bax and Cleaved‐Caspase3 (*P* < .05) (Figure 2D). Results from TUNEL staining indicated that the apoptosis of HK‐1 cells was enhanced by MEG3 overexpression (*P* < .05) (Figure 2E). Taken together, our data exhibited that MEG3 overexpression promoted the autophagy and apoptosis of NPC cells.

### 3.3 MEG3 increases PTEN expression by binding to miR‐21

**FIGURE 3:**
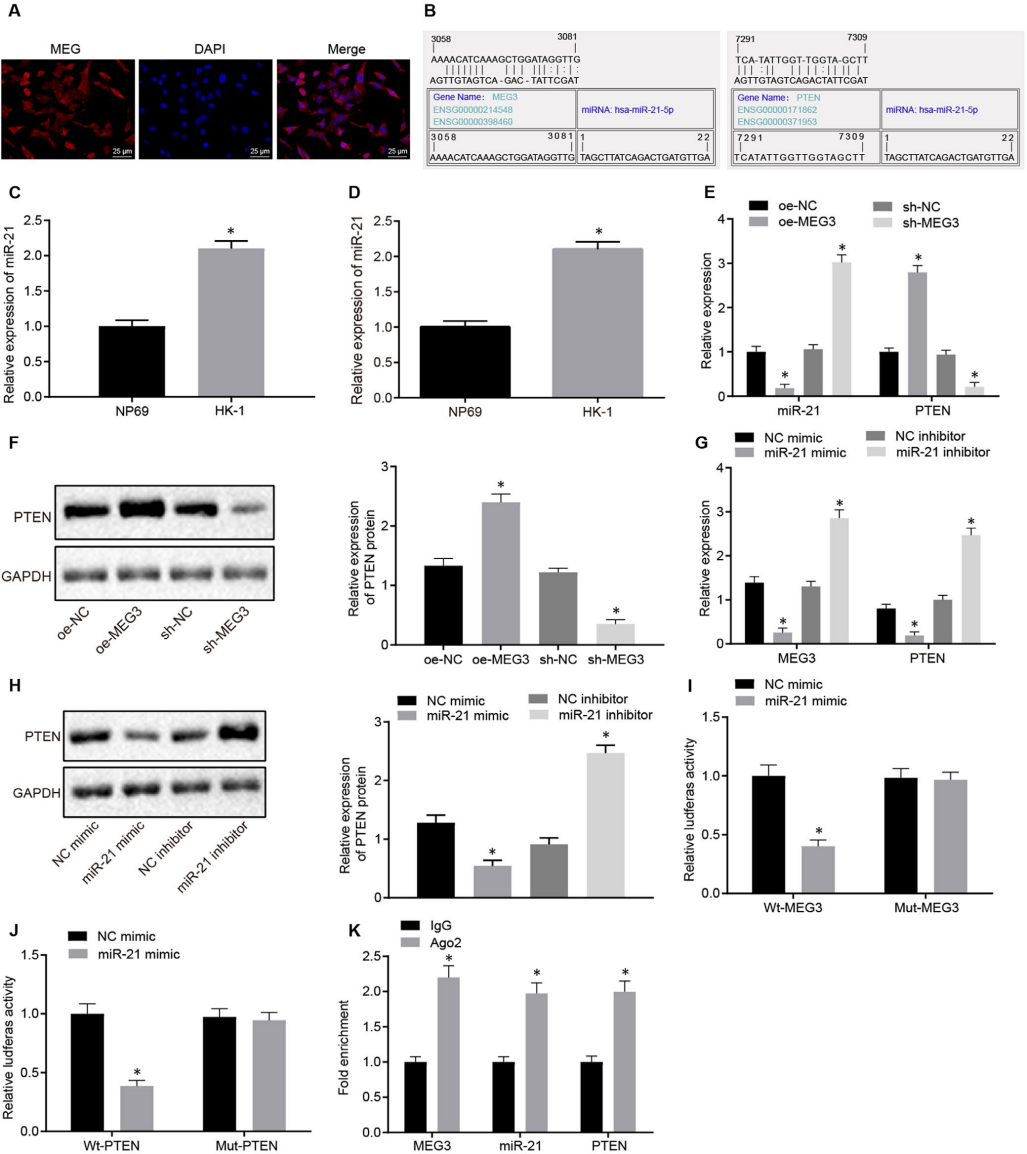
The expression of PTEN was up‐regulated by MEG3 *via* sponging miR‐21. A, Subcellular localization of MEG3 detected by FISH (×400). B, Binding sites of miR‐21 on MEG3 as well as PTEN predicted through the bioinformatics analysis. C, Relative expression of miR‐21 in NPC tissues and paracancerous tissues. D, The level of miR‐21 in NPC cells and normal nasopharyngeal epithelial cells. E, Relative expression of miR‐21 and PTEN in HK‐1 cells treated with oe‐NC, sh‐NC, oe‐MEG3 and sh‐MEG3. F, Western blot analysis of PTEN protein in HK‐1 cells treated with oe‐NC, sh‐NC, oe‐MEG3 and sh‐MEG3. G, Relative expression of MEG3 and PTEN in HK‐1 cells treated with NC mimic, NC inhibitor, miR‐21 mimic and miR‐21 inhibitor. H, Western blot analysis of PTEN protein in HK‐1 cells treated with NC mimic, NC inhibitor, miR‐21 mimic and miR‐21 inhibitor. I&J, Dual‐luciferase reporter gene assay identified a binding relationship between miR‐21 and MEG3, as well as miR‐21 and PTEN. K, Quantification of fold enrichment of MEG3, miR‐21 and PTEN in cells probed with IgG or Ago2 detected by RIP. *P < .05 vs. paracancerous tissues, NP69 cells, HK‐1 cells treated with oe‐NC/sh‐NC/NC mimic/NC inhibitor or cells probed with IgG. The data were described as mean ± standard deviation. An unpaired t test was used to test the comparison between two groups. Three independent experiments were performed

To dissect the possible mechanism underlying MEG3 on NPC development, RNA‐FISH was performed to detect the subcellular localization of MEG3 and identified that MEG3 mainly expressed in the cytoplasm of HK‐1 cells (Figure 3A). Binding sites between MEG3 and miR‐21, miR‐21 and PTEN were predicted on an available bioinformatics database (Figure 3B). The potential binding between MEG3, miR21 and PTEN implies that MEG3 might regulate the expression of miR21 and/or PTEN. miR‐21 was increased in NPC tumour tissues and NPC cells (*P* < .05) (Figure 3C&D). Consistent with the hypothesis, miR‐21 expression was increased in cells after MEG3 knockdown (sh‐MEG3). It was subsequently verified that MEG3 overexpression in HK‐1 cells markedly reduced the miR‐21 expression while up‐regulating the expression of PTEN (Figure 3E).

Thereafter we set to determine the regulation of PTEN expression by MEG3 while the miR‐21 level was further elucidated. Western blot analysis indicated an increased level of PTEN protein in HK‐1 cells expressing MEG3 (*P* < .05) whereas the PTEN level was reduced in HK‐1 cells with MEG3 knocked down (Figure 3F). These results suggested that MEG3 negatively regulated PTEN expression. By conducting similar gain‐ and loss‐of‐function assays, we found that miR‐21 could positively regulate the PTEN expression (Figure 3G&H).

The HK‐1 cells were cotransfected with NC mimic or miR‐21 mimic with wt‐MEG3, mut‐MEG3, wt‐PTEN and mut‐PTEN, respectively. The result showed decreased fluorescence intensity in cells cotransfected miR‐21 mimic with wt‐MEG and wt‐PTEN (*P* < .05), compared with cells transfected with NC mimic. In contrast, HK‐1 cells co‐expressing miR‐21 mimic with mut‐MEG3 and mut‐PTEN showed no difference (*P* > .05) (Figure 3I&J). Interestingly, the binding of miR‐21 to MEG3 and PTEN was detected by RIP assay, which implied that the combination of miR‐21 with MEG3 and PTEN in cells treated with anti‐Ago2 was significantly increased as compared to the cells treated with anti‐IgG (*P* < .05) (Figure 3K).

### 3.4 MEG3 exerts a stimulative effect on apoptosis and autophagy of NPC cells by inhibiting the miR‐21

**FIGURE 4:**
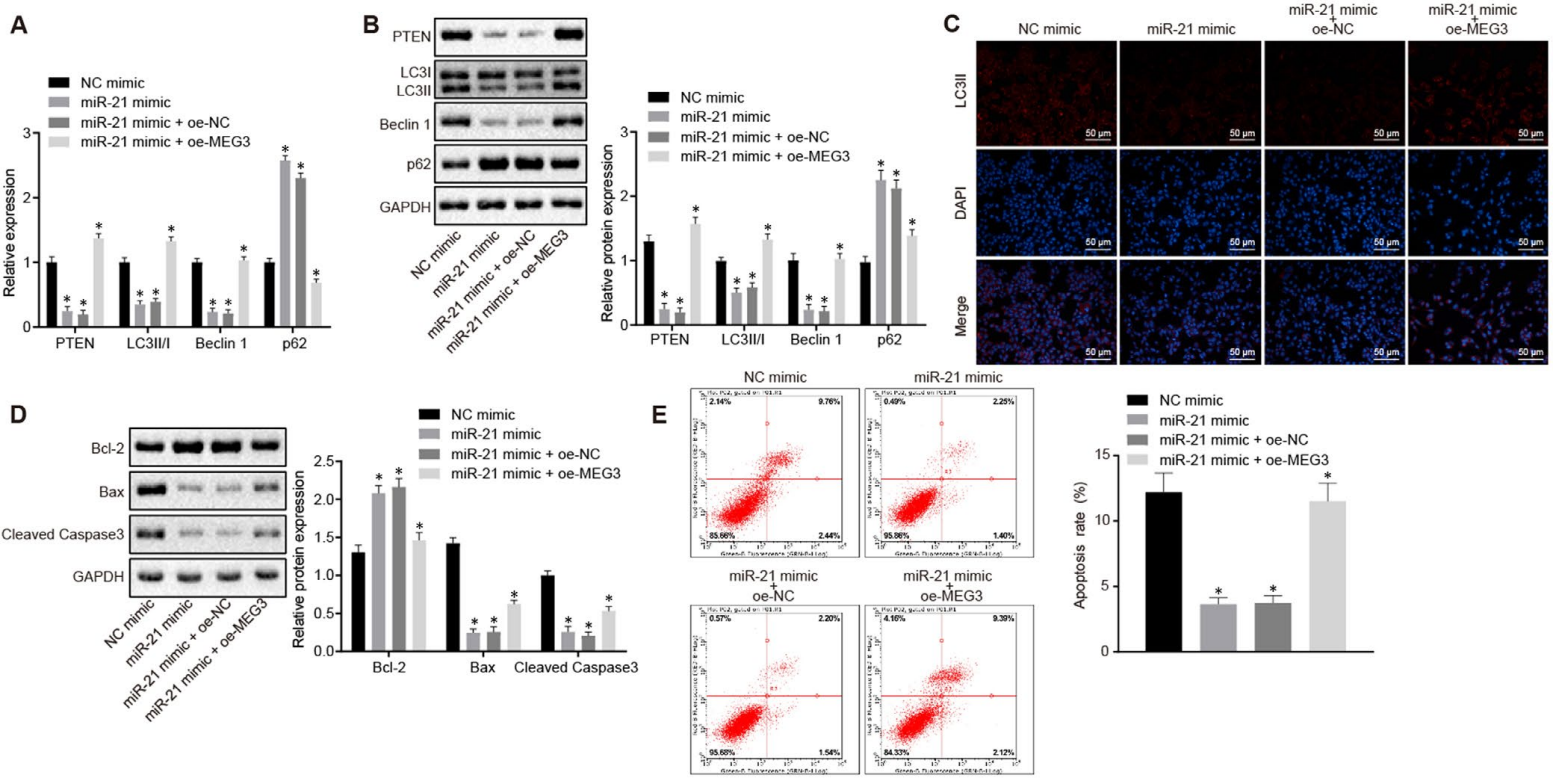
MEG3 down‐regulated miR‐21 to stimulate the apoptosis and autophagy of NPC cells. In this experiment, HK‐1 cells were treated with NC mimic, miR‐21 mimic, miR‐21 mimic plus oe‐NC and miR‐21 mimic plus oe‐MEG3. A, Relative expression of PTEN, LC3 II/I, Beclin1 and P62 in HK‐1 cells after different treatments. B, Western blot analysis of PTEN, LC3 II/I, Beclin1 and P62 proteins in HK‐1 cells after different treatments. C, Immunofluorescence image of LC3 II/I in HK‐1 cells after different treatments (×200). D, Western blot analysis of Cleaved‐Caspase3, Bcl‐2, Bax and GAPDH proteins in HK‐1 cells with different treatments. E, Flow cytometry analysis of apoptosis and apoptosis rate in HK‐1 cells after different treatments. *P < .05 vs. cells treated with the NC mimic or miR‐21 mimic plus oe‐NC. The measurement data were described as mean ± standard deviation. An unpaired t test was used to test the comparison between the two groups. Three independent experiments were performed

HK‐1 cells treated with miR‐21 mimic exhibited an increase in the P62 expression and a reduction in the PTEN, LC3II/I and Beclin1 expression (*P* < .05), compared with HK‐1 cells treated with NC mimic. In contrast to the HK‐1 cells transfected with miR‐21 mimic, increased expression of PTEN, LC3II/I and Beclin1 as well as a reduction in expression of P62 was observed in HK‐1 cells cotransfected with miR‐21 mimic and oe‐MEG3 (*P* < .05) (Figure 4A&B). Furthermore, the immunofluorescence assay showed the lower intensity of LC3 II/I in HK‐1 cells treated with miR‐21 mimic than cells treated with NC mimic (*P* < .05), however, a higher level of LC3 II/I was observed in the HK‐1 cells treated with miR‐21 mimic plus oe‐MEG3 than the HK‐1 cells treated with miR‐21 mimic plus oe‐NC (*P* < .05) (Figure 4C). In summary, results shown here indicated that miR‐21 overexpression inhibited PTEN expression and the autophagy of HK‐1 cells. Moreover, it also suggested that MEG3 expression can reverse the inhibitory effect of miR‐21 on PTEN as well as the autophagy of HK‐1 cells.

To investigate the effects of MEG3 and miR‐21 on apoptosis of HK‐1 cells, the expression of apoptosis‐related proteins, that is Cleaved‐Caspase3/Bcl‐2/Bax in HK‐1 cells treated with NC mimic, miR‐21 mimic, miR‐21 mimic plus oe‐NC and miR‐21 mimic plus oe‐MEG3 was measured using RT‐qPCR and Western blot analysis. Compared to cells treated with NC mimic, the expression of Bcl‐2 protein was increased while the Cleaved‐Caspase 3 and Bax were decreased in cells treated with miR‐21 mimic (*P* < .05), suggesting that up‐regulated miR‐21 can inhibit apoptosis of HK‐1 cell. In contrast, reversed changes in those apoptosis‐related proteins were identified in cells treated with miR‐21 mimic plus oe‐MEG3, compared to cells treated with miR‐21 mimic plus oe‐NC (Figure 4D). Hence, these findings suggested that highly expressed MEG3 can reverse the inhibitory effect of miR‐21 on the apoptosis of HK‐1 cells, which were further verified by flow cytometric data (Figure 4E).

### 3.5 MEG3 promoted the autophagy and apoptosis of NPC cells by increasing the expression of PTEN

**FIGURE 5:**
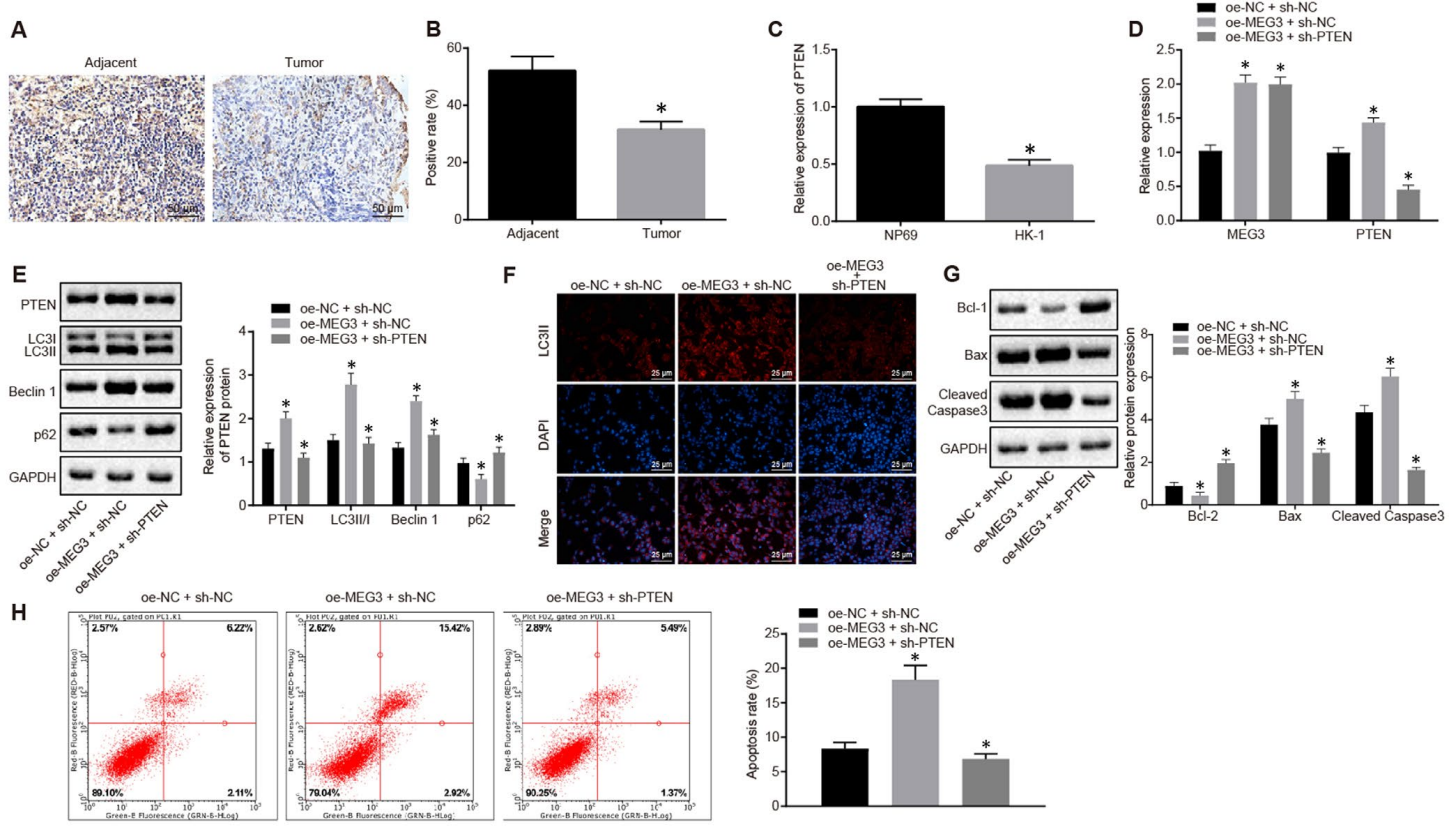
The autophagy and apoptosis of NPC cells were promoted by highly expressed MEG3 and PTEN. A&B, Relative expression of PTEN in NPC tissues and paracancerous tissues measured using immunohistochemistry and quantitative analysis of PTEN‐positive rate. C, Relative expression of PTEN in NPC cells and normal nasopharyngeal epithelial cells. D, Relative expression of MEG3 and PTEN in HK‐1 cells treated with oe‐NC plus sh‐NC, oe‐MEG3 plus sh‐NC and oe‐MEG3 plus sh‐PTEN. E, Protein levels of PTEN, LC3II/I, Beclin1, P62 and GAPDH in HK‐1 cells treated with oe‐NC plus sh‐NC, oe‐MEG3 plus sh‐NC and oe‐MEG3 plus sh‐PTEN. F, Immunofluorescence image of LC3Ⅱ (×200). G, Western blot analysis of Cleaved‐Caspase3, Bcl‐2, Bax and GAPDH and their relative expression in HK‐1 cells treated with oe‐NC plus sh‐NC, oe‐MEG3 plus sh‐NC and oe‐MEG3 plus sh‐PTEN. H, Flow cytometric analysis of apoptosis rate in HK‐1 cells treated with oe‐NC plus sh‐NC, oe‐MEG3 plus sh‐NC and oe‐MEG3 plus sh‐PTEN. *P < .05 vs. paracancerous tissues, NP69 cells, HK‐1 cells treated with oe‐NC plus sh‐NC or oe‐MEG3 plus sh‐NC. The measurement data were described as mean ± standard deviation. Independent sample t test was used to compare data between two groups, while one‐way ANOVA and Tukey's post hoc tests were applied for data among multiple groups. Three independent experiments were performed. The difference was defined as statistically significant when *P* < .05

Immunohistochemical staining exhibited a higher PTEN expression in NPC tissues than paracancerous tissues (*P* < .05) (Figure 5A&B). The expression of PTEN in normal nasopharyngeal epithelial cells NP69 and NPC cell line HK‐1 was monitored by RT‐qPCR, which showed that PTEN was poorly expressed in HK‐1 cells relative to NP69 cells (*P* < .05) (Figure 5C).

To elucidate the roles of MEG3 and PTEN in autophagy and apoptosis of NPC cells, HK‐1 cells were treated with oe‐NC plus sh‐NC, oe‐MEG3 plus sh‐NC and oe‐MEG3 plus sh‐PTEN, followed by RT‐qPCR and Western blot analyses, respectively. The cells treated with oe‐MEG3 plus sh‐NC exhibited decreased expression of P62 and increased expression of PTEN, LC3II/I and Beclin1, compared with cells treated with oe‐NC plus sh‐NC (all *P* < .05). In contrast, sh‐PTEN reversed the changes in the levels of P62, PTEN, LC3II/I and Beclin1 caused by oe‐MEG3 (Figure 5D&E). Subsequently, the LC3II protein level in cells treated with different plasmids was detected by immunofluorescence assay. The fluorescence intensity of LC3II was increased in cells treated with oe‐MEG3 plus sh‐NC, compared with cells treated with oe‐NC plus sh‐NC (*P* < .05). On the contrary, LC3II fluorescence intensity was decreased in cells treated with oe‐MEG3 plus sh‐PTEN, compared with cells treated with oe‐MEG3 plus sh‐NC (*P* < .05) (Figure 5F). These results suggested that the autophagy of NPC cells could be promoted by increasing the MEG3 expression, however, it can be reversed by decreasing the PTEN.

Moreover, the stimulative role of MEG3 and inhibitory role of PTEN in the apoptosis of NPC cells were further identified by Western blot analysis (Figure 5G) and flow cytometry (Figure 5H). The pro‐apoptotic effect of MEG3 was reversed by PTEN knockdown.

### 3.6 MEG3 overexpression inhibits oncogenicity of NPC cells in vivo

**FIGURE 6:**
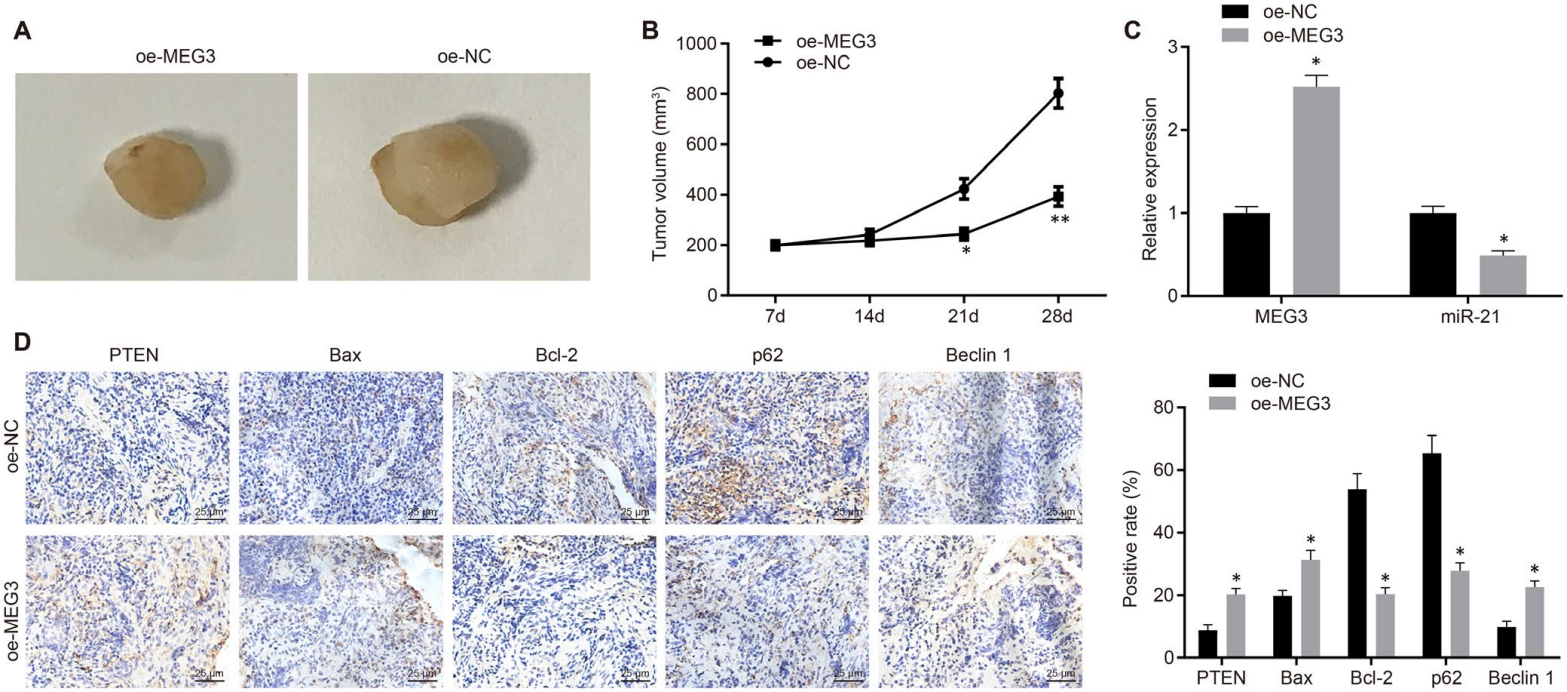
MGE3 acts as a tumour suppressor in NPC development. A, Xenograft tumours from nude mice injected with NPC cells stably transfected with oe‐NC or oe‐MEG3. B, Tumour growth curve for xenograft tumours from nude mice. C, Relative expression of MEG3 and miR‐21 in xenograft tumours detected by RT‐qPCR. D, Immunohistochemical analysis of PTEN, Bax, Bcl‐2, p62 and Beclin1 in xenograft tumours (×400). *P < .05 vs. xenograft tumours from nude mice injected with oe‐NC‐transfected cells. The measurement data were described as mean ± standard deviation. An independent sample t test was used to compare data between two groups. Data of different groups at different time points were compared by repeated‐measures ANOVA and subjected to Bonferroni's post hoc test. n = 12

In vivo experiments were conducted to verify the function of MEG3, which showed that a smaller volume of xenograft tumours and slower tumour growth for nude mice injected with the NPC cells stably transfected with oe‐MEG3 (*P* < .05) (Figure 6A&B). RT‐qPCR identified a higher MEG3 expression and lower miR‐21 expression in xenograft tumours from nude mice injected with the oe‐MEG3‐transfected NPC cells, as compared to nude mice with the oe‐NC‐transfected NPC cells (*P* < .05) (Figure 6C). Additionally, PTEN, Bax and Beclin1 protein levels were up‐regulated whereas Bcl‐2 and p62 protein levels were down‐regulated in xenograft tumours from nude mice when MEG3 was overexpressed (*P* < .05) (Figure 6D). Hence, these above‐reported results indicated that the oncogenicity of NPC cells in nude mice was inhibited by MEG3 overexpression.

## 4 DISCUSSION

Based on the status quo, targeted therapy provides a promising opportunity for NPC treatment.^3^ Highly expressed MEG3 has been proven to play a suppressive role in proliferation and migration of glioma cells^14^ as well as promoting apoptosis and inhibiting differentiation of ovarian cancer cells.^15^ Intriguingly, our data indicated the poor expression of MEG3 in NPC tissues and cells whereas overexpression of MEG3 was suggested to facilitate the autophagy and apoptosis of NPC cells by impairing the miR‐21‐mediated down‐regulation of PTEN.

Initially, our results exhibited that restoration of MEG3 promoted the apoptosis and autophagy of NPC cells. Nevertheless, accumulating studies have reported the pro‐apoptotic and pro‐autophagic effects of MEG3 on cancer cells in various human cancers. For instance, MEG3 impeded cell growth while promoting apoptosis and autophagy in glioma cells.^14^ Moreover, MEG3 contributed to the promotion of cell apoptosis in non‐small cell lung cancer (NSCLC).^16^ Additionally, recent studies have demonstrated the pro‐apoptotic effect of MEG3 on head and neck cancers. For instance, ectopic expression of MEG3 could accelerate the cell apoptosis in oesophageal cancer.^17^ MEG3 has been reported to induce the apoptosis of laryngeal cancer cells *via* activation of caspase‐9 and caspase‐3.^18^ Furthermore, MEG3 was suggested to play tumour‐suppressive role in oral squamous cell carcinoma by impeding cell proliferation.^19^ Consistently, results in our study further supported the pro‐apoptotic role of MEG3 in NPC. In addition to this role, a pro‐autophagic role of MEG3 was also supported by the up‐regulation of LC3II and Beclin1, and down‐regulation of p62. Results from another study briefly elucidated the inhibitory effect of MEG3 on in vivo tumorigenicity of NPC cells.^8^ In the present study, the tumour‐suppressive role of MEG3 was further verified by in vivo assays. MEG3 can function as a competitive endogenous RNA, to mediate the key signalling proteins by which MEG3 coordinates a series of cellular processes protecting against cancer or metabolic disorders.^20^ Thus, we further aimed to investigate the downstream mechanism of MEG3 in this malignancy.

Furthermore, we experimentally identified the binding relationship between MEG3 and miR‐21 as well as PTEN and miR‐21. Our results exhibited that the expression of miR‐21 was up‐regulated whereas the expression of PTEN was down‐regulated in NPC cells. Interestingly, aberrantly high expression of miR‐21 has been reported in tissues and cell lines of various human cancers such as gastric cancer and breast cancer, respectively.^21^, ^22^ Moreover, it has been documented that PTEN was down‐regulated by abundantly expressed EBV‐miR‐BART7‐3p in NPC.^23^ PTEN has been characterized as the target gene of miR‐21 and highly expressed miR‐21 leads to a reduction in the PTEN expression^24^. Similarly, experiments in a recent study demonstrated a crosstalk between miR‐1297 and MEG3 and a binding of miR‐1297 to PTEN in its 3’‐UTR region.^25^ The most crucial findings of our study demonstrated that MEG3 promoted apoptosis and autophagy of NPC cells *via* up‐regulating PTEN by binding to miR‐21. It has been indicated that in gastric cancer cells, MEG3 suppressed the cell migration and invasion by down‐regulating the expression of miR‐21, whereas up‐regulated miR‐21 counteracted the suppressive effect of MEG3 on cell mobility.^26^ Consistently, another study has illustrated the role of PTEN as a tumour suppressor gene in NPC, however, miR‐21 increases the proliferation and reduces the apoptosis of NPC cells by inhibiting PTEN.^27^ Collectively, these above‐reported findings support the inference that interaction of MEG3, miR‐21 and PTEN could be the key mechanism in the regulation of cellular processes in NPC. Although the anti‐autophagic function of miR‐21 has been elaborated in the hypoxia/reoxygenation‐exposed cardiomyocytes,^28^ and renal tubular epithelial cells during renal ischaemia‐reperfusion,^29^ its involvement in the human cancer cells remains largely unknown. miR‐21 was recently proposed as an inhibitor of autophagy in gastric cancer and therefore to enhance drug resistance.^30^ The present study emphasized the tumour‐promotive role of miR‐21 in NPC by suppressing the cell autophagy. Notably, PTEN has been reported to be frequently mutated in human cancers and is a well‐known anti‐oncogene that encodes a dual‐specificity phosphatase antagonizing the phosphatidylinositol 3‐kinase (PI3K) class I/AKT/mTOR pathway.^31^ mTOR is a key checkpoint that negatively regulates the autophagy and inhibition of the PI3K/AKT/mTOR pathway potentially stimulates autophagy for prevention of cancer progression.^32^ Considering the close relationship between PTEN and autophagy, our study further substantiated that PTEN knockdown reversed the promotive effect of MEG3 on NPC cell autophagy and apoptosis. Those data suggested the importance significance of a MEG3/miR‐21/PTEN axis in the regulation of autophagy and apoptosis in NPC cells.

## 5 CONCLUSION

**FIGURE 7:**
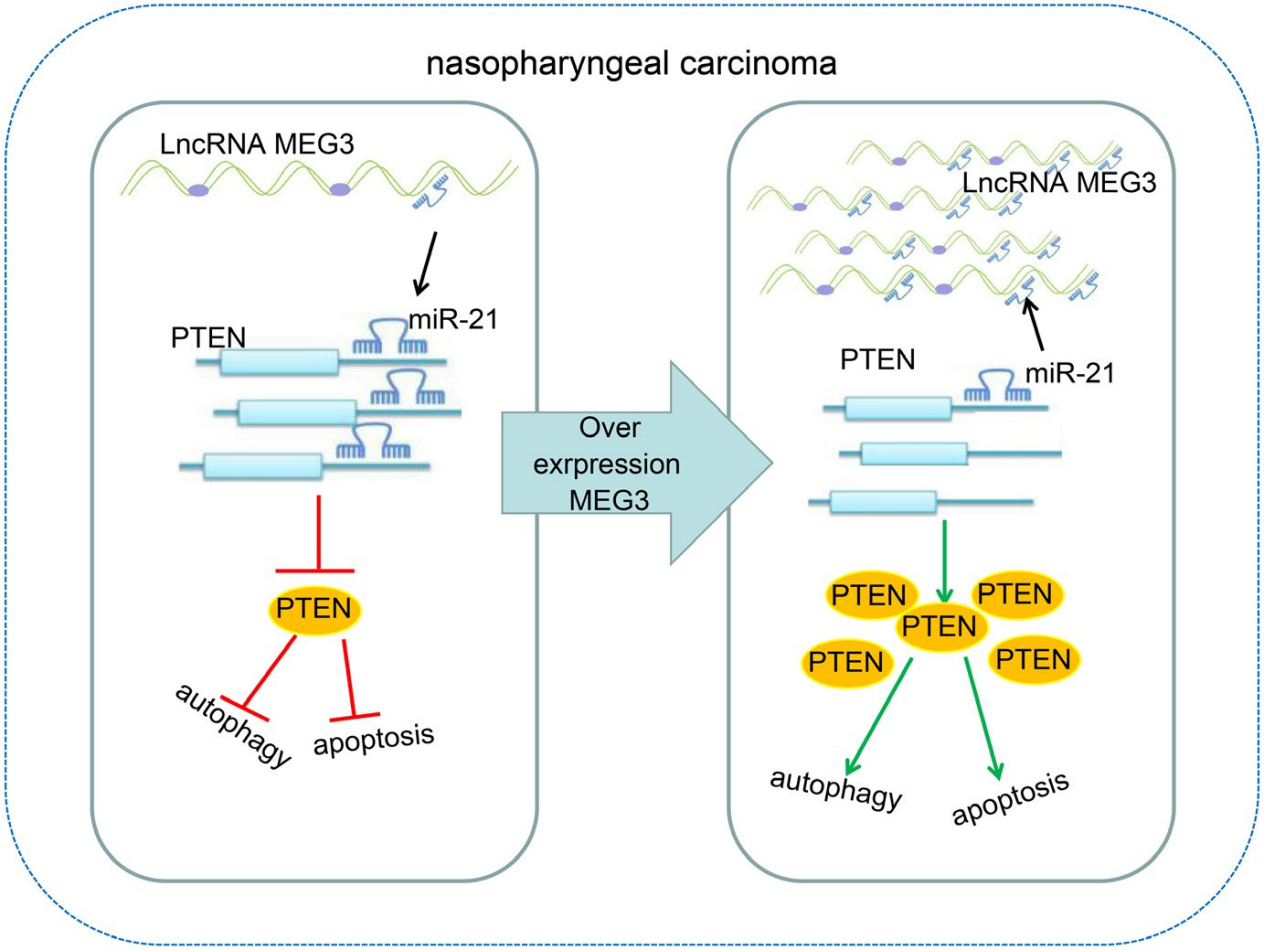
MEG3 overexpression can promote autophagy and apoptosis of NPC cells by increasing PTEN expression through interacting with miR‐21

In summary, MEG3 acts as a tumour suppressor in NPC that can promote the autophagy and apoptosis of NPC cells *via* increasing PTEN through interaction with miR‐21 (Figure 7). The findings of MEG3‐mediated autophagy and apoptosis in NPC aid in a better understanding of the in‐depth mechanisms, which will shed light on the important therapeutic implications in NPC. Further experiments on the translation of those findings into clinical practice for NPC treatment are recommended for the development of novel therapeutics.

## CONFLICT OF INTEREST

The authors confirm that there are no conflicts of interest.

## AUTHOR CONTRIBUTIONS

Baotao Lv designed the study. Liqiang Lin and Xiaoli Liu: Data collation, data analyses and initial draft of the manuscript. Baotao Lv: Drafting of the manuscript. All authors have read and approved the final submitted manuscript.

## ETHICAL APPROVAL

The study protocol was approved by the Ethics Committee and Experimental Animal Ethics of Linyi People's Hospital. Informed written consent was obtained from each patient before the study. All the experiments were conducted strictly in accordance with the Helsinki Declaration. The animal experiment strictly adhered to the principle to minimize the pain, suffering and discomfort to experimental animals.

## CONSENT FOR PUBLICATION

Not applicable.

## Data Availability

The datasets generated/analysed during the current study are available.
